# The influence of near-field fluxes on seasonal carbon dioxide enhancements: results from the Indianapolis Flux Experiment (INFLUX)

**DOI:** 10.1186/s13021-020-00166-z

**Published:** 2021-01-30

**Authors:** Natasha L. Miles, Kenneth J. Davis, Scott J. Richardson, Thomas Lauvaux, Douglas K. Martins, A. J. Deng, Nikolay Balashov, Kevin R. Gurney, Jianming Liang, Geoff Roest, Jonathan A. Wang, Jocelyn C. Turnbull

**Affiliations:** 1 Department of Meteorology and Atmospheric Science, The Pennsylvania State University, University Park, PA 16802 USA; 2 Earth and Environmental Systems Institute, The Pennsylvania State University, University Park, PA 16802 USA; 3 Present Address: Laboratoire des Sciences du Climat et de l’Environnement (LSCE), 91190 Saint-Aubin, France; 4 Present Address: FLIR Systems, Inc, West Lafayette, IN 47906 USA; 5 Present Address: Utopus Insights, Inc, Valhalla, NY 10595 USA; 6 Present Address: NASA Goddard Space Flight Center/Universities Space Research Association, Greenbelt, MD 20771 USA; 7 Northern Arizona University, Flagstaff, AZ 86011 USA; 8 Present Address: Environmental Systems Research Institute, Redlands, CA 92373 USA; 9 Boston University, Boston, MA 02215 USA; 10 Present Address: University of California, Irvine, CA 92697 USA; 11 GNS Science, Lower Hutt, 5040 New Zealand; 12 CIRES, University of Colorado at Boulder, Boulder, CO USA

**Keywords:** Carbon dioxide, Urban, Greenhouse gas, Fluxes, Background, INFLUX, Anthropogenic, Biogenic

## Abstract

**Background:**

Networks of tower-based CO_2_ mole fraction sensors have been deployed by various groups in and around cities across the world to quantify anthropogenic CO_2_ emissions from metropolitan areas. A critical aspect in these approaches is the separation of atmospheric signatures from distant sources and sinks (i.e., the background) from local emissions and biogenic fluxes. We examined CO_2_ enhancements compared to forested and agricultural background towers in Indianapolis, Indiana, USA, as a function of season and compared them to modeled results, as a part of the Indianapolis Flux (INFLUX) project.

**Results:**

At the INFLUX urban tower sites, daytime growing season enhancement on a monthly timescale was up to 4.3–6.5 ppm, 2.6 times as large as those in the dormant season, on average. The enhancement differed significantly depending on choice of background and time of year, being 2.8 ppm higher in June and 1.8 ppm lower in August using a forested background tower compared to an agricultural background tower. A prediction based on land cover and observed CO_2_ fluxes showed that differences in phenology and drawdown intensities drove measured differences in enhancements. Forward modelled CO_2_ enhancements using fossil fuel and biogenic fluxes indicated growing season model-data mismatch of 1.1 ± 1.7 ppm for the agricultural background and 2.1 ± 0.5 ppm for the forested background, corresponding to 25–29% of the modelled CO_2_ enhancements. The model-data total CO_2_ mismatch during the dormant season was low, − 0.1 ± 0.5 ppm.

**Conclusions:**

Because growing season biogenic fluxes at the background towers are large, the urban enhancements must be disentangled from the biogenic signal, and growing season increases in CO_2_ enhancement could be misinterpreted as increased anthropogenic fluxes if the background ecosystem CO_2_ drawdown is not considered. The magnitude and timing of enhancements depend on the land cover type and net fluxes surrounding each background tower, so a simple box model is not appropriate for interpretation of these data. Quantification of the seasonality and magnitude of the biological fluxes in the study region using high-resolution and detailed biogenic models is necessary for the interpretation of tower-based urban CO_2_ networks for cities with significant vegetation.

## Background

The ability to accurately and annually quantify urban greenhouse gas (GHG) emissions is essential for assessing the interim effectiveness of decadal-scale urban climate action plans (e.g., [1]). Each of numerous approaches to estimating urban CO_2_ emissions has strengths and weaknesses, and the approaches are ideally used synergistically to improve each other [2]. An advantage of in situ tower- and building top- based atmospheric approaches is the ability to provide continuous quantification of emissions to support urban policy mitigation plans. Consequently, an increasing number of such GHG monitoring networks have been deployed in cities around the globe (e.g., [3–8].

The choice of background is critical for interpretation of data from urban CO_2_ mole fraction networks because of the need to isolate the urban signal from variations associated with weather [9, 10] and sources and sinks from other locations. The effect of background choices is an active area of research. Lauvaux et al. [11] used two-step optimization of the boundary conditions, first utilizing aircraft and CarbonTracker inverse system [12] mole fractions, and then optimizing within the inversion. McKain et al. [4] used CO_2_ mole fractions from a mountaintop site to represent the background CO_2_, as did Lauvaux et al. [13]. Verhulst et al. [8] analyzed four potential background sites for Los Angeles, California, and determined the annual average uncertainty in background using a local marine site to be roughly 10% of the median mid-afternoon CO_2_ enhancement for their 50-m AGL rooftop measurement site near downtown. For the CO_2_-MEGAPARIS experiment, also described by Bréon et al. [14] and Xueref-Remy et al. [15], Staufer et al. [7] used three CO_2_ measurement sites in a Lagrangian configuration, with the background used depending on strictly defined wind speed and wind direction parameters. The site used as background for northeasterly winds was located in a small village considered a rural area, and the background site for southwesterly winds was near the southwest corner of Paris, France, in a mixed urban and rural area. Sargent et al. [16] calculated a curtain of background values using data from two background sites 90–170 km from Boston, Massachusetts, combined with modelled vertical mole fraction gradients, and limited the analysis to days with wind directions within ± 40° of the background to urban site vector. CO_2_ for each edge of the model domain boundaries was determined by Nickless et al. [6] using a Global Atmosphere Watch (GAW) station located 60 km to the south of Cape Town, South Africa. The determination of background for Cape Town was aided by its location on a peninsula. Mueller et al. [17] used modeling and geostatistical methods with synthetic data to determine optimal locations for four background towers in the Washington D.C./Baltimore area. Cities predominately downwind of large bodies of water or located in non-vegetated regions are simpler in terms of determination of background, but most cities, including those described above, are near other cities and/or surrounded by active vegetation, complicating the extraction of local signals.

The Indianapolis Flux Project (INFLUX) is a testbed for measuring urban GHG emissions in Indianapolis, Indiana [2, 18]. For the dormant season in Indianapolis, roughly November–March, the biogenic effect on CO_2_ fluxes is relatively small compared to the fossil fuel contribution. Turnbull et al. [19] found that wintertime CO_2_ enhancement in Indianapolis was nearly equivalent to the fossil-fuel CO_2_ enhancement using a local background. Also in Indianapolis, Miles et al. [5] used a single predominantly upwind tower site 20 km to the southwest of the city edge as background for an analysis of inter-tower differences during the dormant season 2012–2013. Lauvaux et al. [20] found that inverse emission results differed by only 4% between using a single background and using a wind direction dependent background, for their primarily dormant season analysis of data between September 2012 and April 2013. Turnbull et al. [21] used flask measurements of ^14^CO_2_ (comparing downwind towers to a local background site (Tower 01)) to determine that biogenic CO_2_ fluxes in Indianapolis are just 10% of the magnitude of fossil fuel CO_2_ fluxes in November and December.

For analyses encompassing the growing season (roughly April–October in the Northern Hemisphere mid-latitudes), and for cities without dormant seasons, however, biogenic contributions to the total flux are potentially substantial and must be addressed [6]. In the growing season, biological fluxes significantly impact the determination of the urban carbon budget [19, 22, 23]. In addition to a strong seasonal cycle in biological CO_2_ fluxes, different vegetation types are known to exhibit different timing of fluxes. For example, crops have shorter growing seasons and more intense carbon drawdown than natural vegetation [24, 25]. Corbin et al. [26] found that simulating corn and soybean explicitly alters both the timing and magnitude of the net carbon fluxes compared to generic agriculture/grassland. Increasing agricultural land use and yields between 1961 and 2010 manifested in a 15% long-term increase in CO_2_ seasonal amplitude and a shift in overall vegetation growth by 1 to 2 weeks [27]. Differences in green-up dates of local vegetation were correlated with associated patterns in CO_2_ in Boston [28]. In the U.S. Upper Midwest, Miles et al. [9] found large growing season mean differences in CO_2_ (5.1 ppm) between a tower site dominated by corn versus one dominated by grass. Comparatively, a downtown tower (Tower 03) in Indianapolis measured 2.9 ppm higher than a forested tower (Tower 01) during the dormant season. Thus biological fluxes can be on the same order of magnitude as anthropogenic fluxes and need to be taken into account when interpreting urban CO_2_ observations.

Evaluation of the impact of background biological fluxes on urban CO_2_ flux estimates has, to date, relied heavily upon simulations of these fluxes. Nickless et al. [6] used the Community Atmosphere Biosphere Land Exchange (CABLE) model to represent biogenic fluxes for their inversion of CO_2_ fluxes in Cape Town, South Africa. While the inversion was able to improve the total flux estimates, it was not able to disaggregate the biogenic from anthropogenic fluxes because of the large uncertainties in the biogenic flux priors. Sargent et al. [16] used the UrbanVPRM and a biomass map to simulate the impact of rural biology on background CO_2_ for the city of Boston. Heimburger et al. [29] and other aircraft studies show that background CO_2_ mole fractions often exhibit complex spatial structure. Balashov et al. [30] show that Indianapolis background for CH_4_, another greenhouse gas produced by various anthropogenic and natural sources, varies in space with large random and systematic differences across background sites, likely attributable to large plumes from coal mines in southwest Indiana. Direct evaluation of background CO_2_ via long-term observation is needed to assess the accuracy of our urban inversion systems.

The goal of this paper is to document the effects of background choice on CO_2_ enhancements above background measured with an urban tower-based network. We first consider the differences between background towers as a function of wind direction and season. Two potential background towers were available for January 2013–December 2018, and an additional one was available for April 2017–December 2018. We calculated 31-day median afternoon enhancements above each of the primary background towers, and composited over 5.7 years of data to determine yearly cycles of enhancements at each tower. We compared the results from each of the background towers, and made a simple prediction to explain the difference between the towers based on their land cover types. We then compare these results to forward model CO_2_ enhancement using fossil fuel and biogenic fluxes.

## Methods

### Study site

**Fig. 1:**
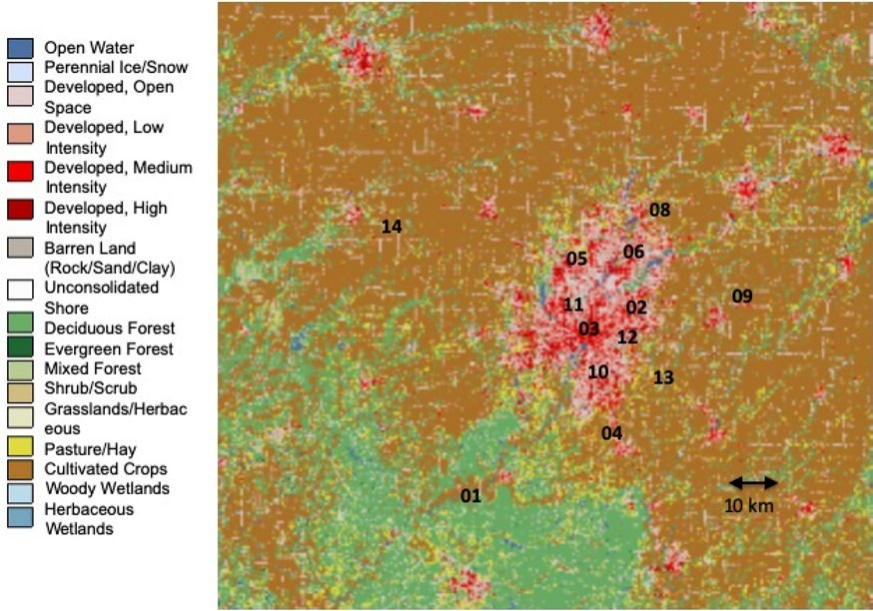
Landcover map of the Indianapolis, IN, region. The numbers 01–14 indicate tower site locations. Towers 05 and 12 were decommissioned in September 2015 and April 2013, respectively. Tower 14 was installed in April 2017 as an additional background site. [33, 34]

The locations of the INFLUX ground-based CO_2_ measurement sites and the city of Indianapolis, Indiana, in the U.S. Midwest, are shown in Fig. 1. Indianapolis, in Marion County, Indiana, was the 17^th^ most populous city in the U.S. in 2019, with an estimated population of about 876,000 [31]. The predominant wind direction in Indianapolis is from the southwest, with southerly through westerly winds occurring 37.7% of the times with appreciable wind speed throughout 2018 [32]. Three of the INFLUX tower sites are considered potential background sites: Towers 01, 09, and 14. Tower 01 is upwind of the edge of the city (as defined by the beltway encircling the city) by 20 km when the wind is from the predominant southwesterly direction, and is on the northern edge of a forested area (Fig. 1). Tower 09 is located in an agricultural area 24 km east of the edge of Indianapolis. Tower 14 was installed in late April 2017, and is northwest of the city by 50 km, again in a primarily agricultural region. The remaining tower sites are in and around Indianapolis [5].

**Fig. 2:**
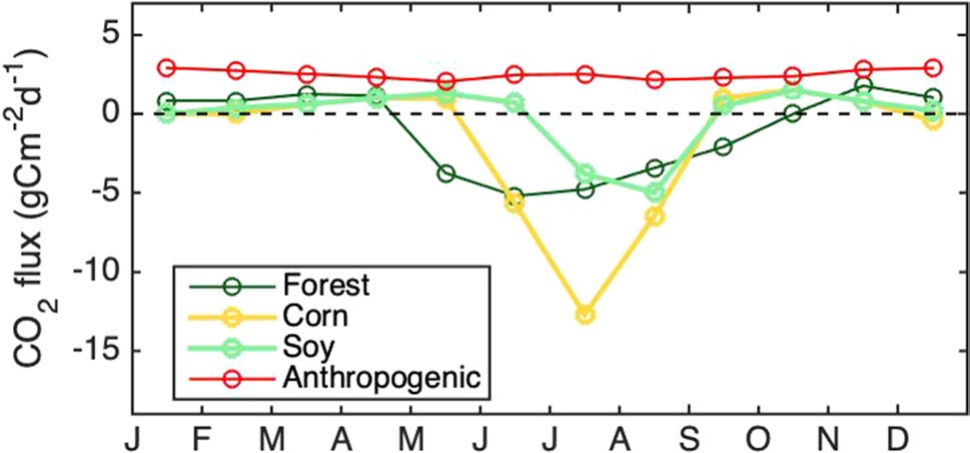
**a** Mean annual cycle of biological CO_2_ fluxes (net ecosystem exchange; NEE) for a forest site (dark green), a corn site (yellow) and a soybean site (light green). The forest fluxes are the 5-year mean measured at the Morgan Monroe State Forest [35] and the corn and soybean fluxes are the 3-year mean measured in Bondville, Illinois [36]. **b** Domain-averaged 31-day median (fossil fuel (Hestia) emissions as a function of time of year for 2014

The primary biogenic land cover types in the region surrounding Indianapolis (Fig. 1) are forest, agricultural (including corn and soy), and grassland/pasture, and the biological fluxes from these land cover types differ significantly in their seasonal cycles (Fig. 2). The forest fluxes shown in Fig. 2 were measured at a flux tower [35] in the Morgan Monroe State Forest (MMSF), 29 km to the south of Tower 01. The monthly average flux is negative in the growing season (i.e., CO_2_ drawdown) beginning in May and extending through September. The forest flux was weakly positive throughout the remainder of the year. A corn/soy flux tower [36] was located in Bondville, Illinois, 176 km WNW of Tower 01. Corn and soy were grown in the field surrounding the flux tower in alternating years. The growing season, as indicated by negative fluxes, for corn begins later than for the forest, in June and extended through August. The soy flux was negative only for the months of July and August. As for the forest, the agricultural fluxes were weakly positive during the dormant season. Grassland/pasture tends to be on the edges of other landcover types in small patches ([5, 37] Fig. 1).

The domain-averaged fossil fuel CO_2_ flux (Hestia, Fig. 2) exhibited higher values in the colder months of November–March, due to energy production for heating (e.g., [7, 13, 38]). The summer increase in fossil fuel emissions was likely attributable to energy production for cooling [39, 40].

### Instrumentation

The INFLUX in situ observation network includes tower sites measuring greenhouse gases using wavelength-scanned cavity ring down spectroscopic (CRDS) instruments (Picarro, Inc., models G1301, G2301, G2302, and G2401). The full network consists of twelve sites but with site re-locations throughout the period, there are a total of fourteen measurement locations. We focus on CO_2_ measured during the period January 2013–December 2018 for this paper. The instruments were deployed at the base of existing communications towers, with ¼″ (0.64 cm) sampling tubes installed as high as possible on each tower (39–136 above ground level (AGL)). Four of the towers had multiple measurement heights (Table 1).

**Table 1:** Measurement height(s) as of 2013 (with 2018 shown in parentheses if different) and predominant landcover type(s) at INFLUX towers

	Predominant landcover type(s)	Latitude (°N)	Longitude (°W)	Measurement height(s) (m AGL)
*Tower 01*	*Forest/agriculture*	*39.5805*	*86.4207*	*10/40/121*
Tower 02	Urban	39.7978	86.0183	10/40/136 (136)
Tower 03	Urban	39.7833	86.1652	10/20/40/54
Tower 04	Urban/agriculture	39.5925	86.1009	60
Tower 05	Urban	39.8947	86.2011	125
Tower 06	Urban	39.9201	86.0280	39
Tower 07	Urban	39.7739	86.2724	58
Tower 08	Agriculture/urban	40.0411	85.9734	41
*Tower 09*	*Agriculture*	*39.8627*	*85.7448*	*10/40/70/130 (130)*
Tower 10	Urban	39.7181	86.1436	40
Tower 11	Urban	39.8403	86.1763	130
Tower 12	Urban	39.7637	86.0403	40
Tower 13	Agriculture	39.7173	85.9417	87
*Tower 14*	*Agriculture*	*39.9971*	*86.7396*	*76*

Additional tower details are listed in Miles et al. [5, 37]

Towers considered as background options are italicized

A linear calibration based on three to five NOAA tertiary reference tanks was applied to the instruments prior to deployment and following any manufacturer repairs, and an intercept-only calibration was applied in the field every 23 h based on one or two calibrated reference tanks. For the majority of the time period, the air samples were dried using Nafion dryers (MD-070-96S-2, PermaPure) in reflux mode, with an internal water vapor correction applied for the effects of the remaining water vapor (< 0.2 or 0.6%, depending on the length of the Nafion dryer). We used hourly means of CO_2_, which were reported on the WMO X2007 scale and are publicly available [5, 37]. Compatibility of the INFLUX network, both within network and compared to the global network, was assessed via co-located NOAA flask systems [41] at Towers 01, 02, 03, 06, 09, and 10, and round-robin type testing using multiple NOAA-calibrated tanks, indicating compatibility of 0.18 ppm CO_2_. Further details of the instrumentation and compatibility are described by Richardson et al. [42] and Miles et al. [5].

Because of unexpectedly low CO_2_ mole fractions measured at Tower 14 during the growing season of 2017, a separate instrument with a separate ¼” (0.64 cm) tube installed to the top of the tower was co-located at the site for a period of several weeks to eliminate the possibility of further leaks or other instrument problems. The additional instrument was calibrated prior to deployment and installed with no drying, relying on the internal water vapor correction for CO_2_. From 20 to 26 August 2018, both instruments sampled from the top of the tower and the difference between the two (primary instrument − secondary instrument) was very small, − 0.11 ± 0.12 ppm.

### Wind measurements

The wind data used to characterize overall synoptic patterns in the city were measured at the Indianapolis International Airport, outside the southwest corner of the city. The data are part of the Integrated Surface Dataset (https://www.ncdc.noaa.gov/isd). The weather station at the airport uses the Automated Surface Observing System (http://www.nws.noaa.gov/asos/pdfs/aum-toc.pdf). The accuracy of wind speed is ± 1.0 ms^−1^ or 5% (whichever is greater) and the accuracy of wind direction is 5° when the wind speed is ≥ 2.6 ms^−1^. Wind directions are not reported for periods in which the wind speed is less than 1.6 ms^−1^. The height of the wind instrument is about 10 m AGL. The wind data are reported at a single point in time recorded within the last 10 min of each hour.

For the purpose of categorizing afternoon-average CO_2_ in terms of wind direction, we calculated vector averages of afternoon winds.

### Determination of land cover surrounding tower locations

In order to characterize each of the INFLUX tower locations in terms of the surrounding land cover types, we considered the land cover within 10 km of each tower. This radius covers approximately 80% of the influence for the towers, determined via afternoon influence functions simulated for January–April 2013 at 1-km resolution with the Lagrangian Particle Dispersion Model (LDPM) [20, 43], using inputs from the Weather Research Forecasting-Four-Dimensional Data Assimilation modeling system (WRF-FDDA-CO_2_) [44–46].

Land cover data was obtained from the United States Department of Agriculture National Agriculture Statistics Service (NASS; [47, 48]). The categories with significant percentages in the Indianapolis area included corn, soy, open water, developed/open space, developed/low intensity, developed/medium intensity, developed/high intensity, deciduous forest, and grass/pasture. The most recent year available from NASS, 2018, was used for the analysis. Corn and soy are typically rotated each year, but since we are considering a large area, a single year is a reasonable representation of the overall landcover for the time period.

We defined the total urban fraction for each tower as the fraction of area categorized as “developed”, compared to the total area within a 10-km radius (80% of the footprint) of each the towers. We then ranked the towers in terms of total urban fraction. Each category of “developed” (i.e., open, low-, medium- and high-intensity) was weighted equally for this purpose, given that the proportions of these categories do not differ considerably between the INFLUX sites.

**Fig. 3:**
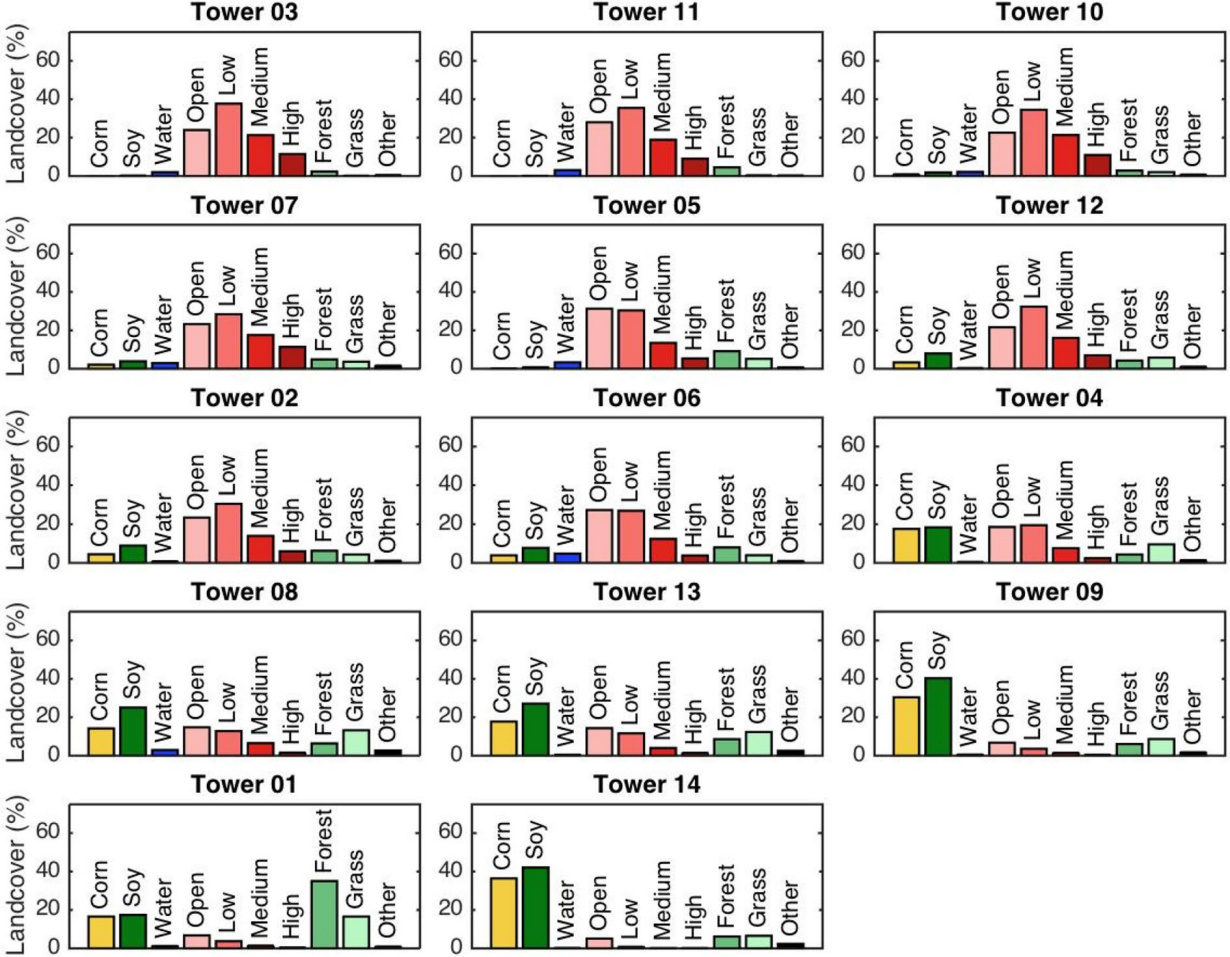
Percentage landcover types for a 10-km radius circle encompassing approximately 80% of the footprint for each tower using the NASS database [47, 48]. The grass category also includes hay/pasture. Towers are ordered based on urban fraction (including open-, low-, medium-, and high-density developed areas). Towers 09, 01, and 14 are potential background towers

The prevalence of different land cover types differed considerably between the INFLUX towers. The percentages of land cover types within each category surrounding each tower are shown in Fig. 3. The towers with the highest urban fractions within the surrounding area were Tower 03 (94.5%), Tower 11 (91.4%), Tower 10 (89.4%), Tower 07 (80.7%), Tower 05 (80.6%), Tower 12 (77.2%), Tower 02 (74.0%), and Tower 06 (70.6%). We consider these “urban” towers. The remaining towers were surrounded by 48% or less urban fraction and are considered “rural” towers. The potential background towers, Towers 09, 01 and 14 were surrounded by 12.3%, 12.3% and 6.2% urban land cover, respectively. Tower 01 was surrounded by the highest fraction of deciduous forest (35.1%). Towers 14 and 09 were primarily agricultural sites (covering 78.5 and 70.7%, respectively, of the surrounding areas). Tower 14 had the highest percentage of corn (36.4%), with Tower 09 at 30.4%. Soy is the other major crop in the region. Note that the radius considered (5, 10, 18 km) for the landcover determination does somewhat affect the ordering of the sites from most urban to least urban, but for all cases, there is a clear grouping between the urban sites (Sites 02, 03, 05, 06, 07, 10, 11, and 12) and the rural sites (01, 04, 08, 09, 13, and 14).

### Observational analyses

In this paper, we focused on afternoon average CO_2_, with the mean calculated over the period 1700–2200 UTC (1200–1700 LST). The atmospheric boundary layer (ABL) is typically well mixed during these hours, which allows simpler interpretation of the measurements (e.g., [49]).

We considered three towers as potential background sites in this paper. Miles et al. [5] used Tower 01 (forested) as the background tower for the INFLUX network during the dormant season, and determined the enhancement in CO_2_ of the other towers compared to it. Here we considered Towers 09 and 14 (both agricultural) as additional background towers. Both Towers 01 and 09 data were available for the majority of the analysis period, January 2013–December 2018, but Tower 14 was only available from April 2017 through December 2018.

We compared the CO_2_ measured at the INFLUX sites by calculating the 31-day running median of the enhancement in CO_2_ for each of the towers from Tower 01 and from Tower 09. Here we use the term enhancement although the difference can be negative, and we note that we have not isolated the anthropogenic enhancement with this calculation. The 31-day running median was chosen to focus on seasonal variability. We excluded data points for which any of the background towers were downwind of the urban area (afternoon average wind directions of 20–65° (Tower 01 in urban plume) and 235–280° (Tower 09 in urban plume). To determine these wind directions, we considered the angle between the background towers and the geometric edges of the urban area, as defined as the region within the expressway encircling Indianapolis (I-465) and the differences between background towers as a function of wind direction in the dormant season. The urban plume was not apparent at Tower 14. Furthermore, median results centered within 10 days of extended data gaps attributable to instrument malfunctions were excluded. We averaged these results for each day of the year for the available years between January 2013 and December 2018 to form annual cycles of smoothed composited CO_2_ differences. The standard deviation of the data from each of the available years was used as an estimate of the variability. We compared the results using Tower 09 as a background to those using Tower 01 as a background. The enhancements at each tower using these two different backgrounds can be compared since the same wind directions were excluded from each. We have primarily approached the background in an Eulerian sense, comparing each tower’s measurements to a background at the same point in time, afternoon averages for this case. We also considered a more Lagrangian approach, utilizing a wind-direction dependent background, with Tower 01 as a background for wind directions from the west (180–360°) and Tower 09 as a background for wind directions from the east (0–180°), as done by Lauvaux et al. [20].

Assuming no missing data, the difference in each tower (Tower N) enhancement using Tower 01 as a background (*CO*_2_,_*Tower N*_ − *CO*_2,*Tower 01*_) and those using Tower 09 as a background *CO*_2_,_*Tower N*_ − *CO*_2,*Tower 09*_ is equivalent to subtracting Tower 09 from Tower 01, since CO_2,*Tower N*_ cancels out. To compare the differences caused by background choice to the enhancement, we normalized the difference in enhancement between using two different background towers by the enhancement using Tower 01 as background and determined the percent difference.

We hypothesize that the differences between Tower 09 and Tower 01 (and thus between the enhancements using these two towers as background) to be attributable to differences in the primary land cover types and corresponding fluxes surrounding these towers. For a measurement site with a finite number of landcover types (*lc*) in the surrounding region, the CO_2_ measured is related to the mean flux from each landcover type (*F*_*lc*_) and the fractional area of that landcover type (*f*_*lc*_),

1 $$CO_{2} = a \mathop \sum \limits_{lc = 1}^{N} F_{lc} f_{lc, }$$

where *a* is a constant. The landcover surrounding Towers 09 and 01 is predominately agricultural and forest, with Tower 09 having a higher percentage of agricultural landcover (71%) and Tower 01 having a higher percentage of forest landcover (35%) and a smaller portion of agricultural landcover (34%). Following from Eq. (1), we assert that,

2 $${\raise0.7ex\hbox{${\Delta CO_{2} }$} \!\mathord{\left/ {\vphantom {{\Delta CO_{2} } a}}\right.\kern-0pt} \!\lower0.7ex\hbox{$a$}} = (F_{forest } f_{forest, 01 } + F_{agr } f_{agr, 01 } ) - (F_{forest } f_{forest, 09 } + F_{ag } f_{agr, 09 } )$$

where ∆*CO*_2_ is the difference in CO_2_ mole fraction between the two tower sites, *F*_*forest*_ and *F*_*agr*_ are the forest and the mean of the corn and soy agricultural fluxes, and *f*_*forest*_ and *f*_agr_ are the forest and agricultural land cover fractions for the area of 10 km radius surrounding each site. Towers 09 and 01 have small and equal amounts of urban landcover (12.3% in the surrounding 10-km radius) so the urban terms cancel. We note that corn and soy are typically rotated from year to year and have similar areal coverage within the region. For these reasons, although corn and soy have very different fluxes, we can simplify the calculation by averaging the corn and soy fluxes to determine an agricultural flux.

This equation assumes that all factors other than the surface fluxes (such as entrainment and advection) are equivalent at the two tower locations. The equation also assumes that the landcover types are not significantly dependent on distance or direction from the tower. This assumption is reasonable for the two towers in Eq. (2) as they are located in homogenous areas, but would not be so for Tower 02, for example, on the downwind edge of the city. We used the forest, corn and soy fluxes shown in Fig. 2a to represent the seasonal pattern of ecosystem flux and predicted midday ABL CO_2_ mole fraction differences between these two background sites. We then compared the seasonal pattern of the predicted CO_2_ difference to the measured differences at Tower 01 and 09.

### Modelled tower CO_2_

We calculated forward modelled CO_2_ dry mole fractions at each of the INFLUX towers by convolving tower footprints, representing the relationship between mole fractions and surface fluxes with fossil fuel emissions and biogenic fluxes. The tower footprints were simulated using transport field derived from the Weather Research and Forecasting model (WRFv3.6.1, [50] in Four-Dimensional Data Assimilation mode for the inner 1-km resolution 87 km × 87 km domain [51]. Data from World Meteorological Organization surface stations within the model domain were assimilated to nudge the model to the observations. The transport fields were then coupled offline to the Lagrangian Particle Dispersion Model [11, 43, 52] in backward mode.

Fossil fuel emissions from the Hestia CO_2_ emissions inventory product [53, 54], available for each of eight economic sectors (residential, on-road mobile, off-road mobile, industrial, commercial, electricity production, airport, and railroad) were used. Hestia emissions were aggregated from the initial building-level product to 1-km resolution, covering Marion County and the eight surrounding counties.

For biogenic fluxes, we used the urban Vegetation Photosynthesis and Respiration Model (VPRM, [55, 56], driven by greenness data from the Moderate Resolution Imaging Spectroradiometer (MODIS) satellite product and climate data from the North America Regional Reanalysis (NARR). The fraction of impervious surface area from the National Land Cover Database [47] within each pixel was used to adjust the carbon fluxes for the impact of urbanization on ecosystem function. Non-paved portions of the city were defined as deciduous broadleaf forest. Distributions for four land cover types (corn, soy, grassland/pasture, and forest) were derived from the United States Department of Agriculture National Agriculture Statistics Service (NASS; [47, 48]), and the VPRM parameters were optimized for these land cover types [57] to produce hourly carbon fluxes at 1-km resolution as the weighted average of carbon fluxes from each type. Note that Tower 14 is outside the domain of the VPRM results and is thus not included in the model-data mismatch analysis.

We then compared the 31-day running median forward modelled CO_2_ enhancements for 2014 to observed CO_2_ enhancements for the same year. We used 1200–1700 LST, with the footprints incorporating fluxes in the 4 h preceding the observations. Wind directions for which either Tower 01 or Tower 09 were within the urban plume were excluded in both the model results and the observations. We then calculated urban site (Towers 02, 03, 06, 07, and 10) averaged model-data mismatch as a function of month of year. Although the percentage of urban landcover surrounding Tower 11 was quite high, we did not include it in the urban site calculations because the forward modelled anthropogenic CO_2_ at Tower 11 was very low.

## Results

### CO_2_ enhancements above background

#### Observed CO_2_ enhancements

**Fig. 4:**
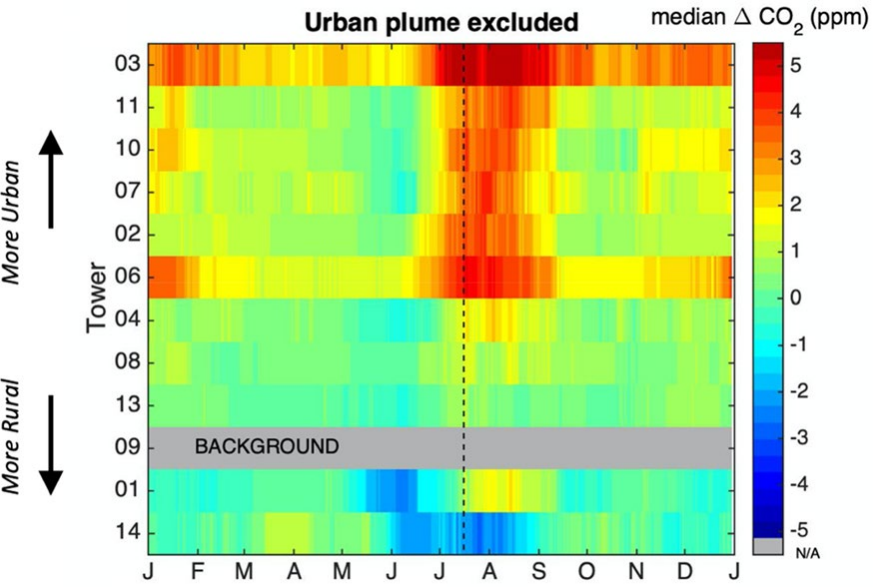
Composited 31-day running median afternoon-average CO_2_ enhancements from Tower 09 for each of the towers, using data from January 2013 through December 2018. The towers are ordered by urban fraction (including high-, medium-, and low-density urban land cover, as discussed in “CO_2_ enhancements above background” section. Tickmarks indicate the beginning of each time period. Data for which Tower 01 or Tower 09 was influenced by the urban plume were excluded from the analysis (WSW and NE). Dashed line indicates July 15. Non-background towers deployed for less than 3 years are not shown (Towers 05 and 12). Tower 09 enhancement compared to Tower 09 is zero, by definition, but the row is included for consistency

When comparing CO_2_ measured at INFLUX towers to a background tower throughout the year, biogenic effects were a dominant feature. The smoothed composited midday ABL CO_2_ mole fraction enhancements relative to Tower 09 (agricultural) are shown in Fig. 4. The overall pattern, consistent for most of the towers, was a maximum in August, a secondary maximum in December–January, and minima in June and October. While anthropogenic fossil fuel CO_2_ emissions have a seasonal pattern (Fig. 2), the intensity of the growing season enhancements is attributable to biogenic effects primarily at the background tower, as will be described.

Not surprisingly, the towers with highest urban fraction in the surrounding area as shown in Fig. 3 exhibited generally higher smoothed composited CO_2_ enhancements relative to agricultural Tower 09 (Fig. 4). There was a clear demarcation between the ‘urban’ towers, those with greater than 70% surrounding urban land cover (Towers 03, 11, 02, 10, 07, and 06), and the ‘rural’ towers with 48% or less urban fraction (Towers 08, 04, 13, 01, and 14). The dominant feature of this figure, the maxima in July/August, was likely attributable to the reduced agricultural and forested land cover within the footprints of the urban towers and thus reduced biogenic uptake of CO_2_ compared to the background tower. In the dormant season, the higher urban tower enhancements were likely due to larger anthropogenic fluxes than for the rural sites. The August enhancements for the urban towers averaged 4.2 ppm, 2.6 times as large as the February urban tower enhancements, which averaged 1.6 ppm. The rural Towers 08, 04, 13, and 01 also exhibited peak growing season maxima compared to Tower 09 but in the range of 1.0–2.5 ppm rather than 4.3–6.5 ppm for the urban towers. The notable difference between Tower 14 and Tower 09 (both agricultural) is discussed in “Agricultural tower CO_2_ background differences” section.

#### Sensitivity of urban CO_2_ enhancement to the choice of background

**Fig. 5:**
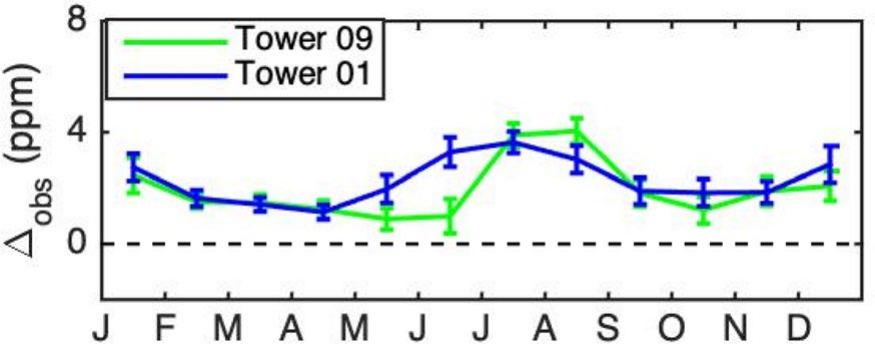
Observed CO_2_ enhancement using Tower 09 (agricultural) as a background (green) and Tower 01 (forested) as a background (blue), composited over 2013–2018, and averaged over INFLUX urban towers. Wind directions for which either Tower 01 or Tower 09 are in the urban plume have been excluded. Only afternoon hours (1200–1700 LST) are included. Error bars indicate the standard error amongst the urban towers

The INFLUX urban tower-average observed enhancements (Towers 02, 03, 06, 07, and 10) were sensitive to the choice of background tower (Fig. 5) during the growing season. While the overall pattern of enhancements (i.e., large summertime enhancement) was evident when comparing to either forested Tower 01 or agricultural Tower 09, the timing of the initiation of the growing season peak differed. The difference in enhancements using the different background towers was largest between May and September, and switched sign in July. In June the difference between using Tower 01 as a background and using Tower 09 as a background was − 2.8 ppm and in August the difference was 1.8 ppm. For the dormant season, the difference in enhancement incurred by using different backgrounds was relatively small, 0.4 ppm on average.

**Fig. 6:**
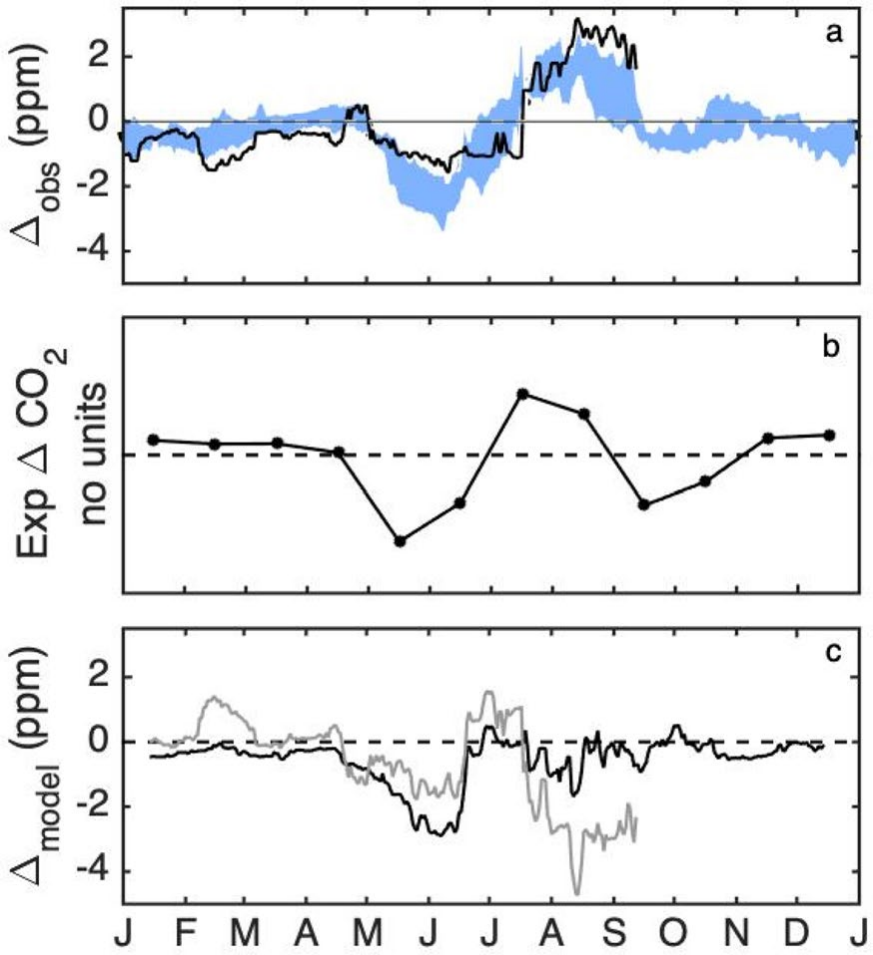
**a** 31-day running median CO_2_ difference between Towers 09 and Tower 01 for 2014 (black line) and composited for 2013–2018 (shaded area), with the width of the shaded area indicating the standard deviation amongst years. Data for which either Tower 01 or Tower 09 were influenced by the urban plume were excluded from the analysis. **b** Predicted seasonal pattern of difference in CO_2_ mole fraction (dimensionless, see Eq. 2) between Tower 01 and Tower 09, based on typical forest, corn and soy fluxes shown in Fig. 2a, and forest and agricultural land cover differences within 10 km of each site, between the two sites. **c** Forward modelled (using Hestia and VPRM) 31-day running median CO_2_ difference (black) between Towers 09 and 01 for 2014. Difference between observed and modelled Tower 01–Tower 09 difference is shown in gray

We postulate that the differences between the enhancements using these two background towers is attributable primarily to the rural biogenic signal. Shown in Fig. 6a is the observed difference between these two background towers, composited over 2013–2018 and for the year for which modelled results are available (2014). The growing season differences between the two towers were stronger in August–September 2014, outside of one standard deviation from the mean of the years, but not as pronounced for May–June. The predicted seasonal pattern CO_2_ difference between Tower 09 and Tower 01, based on forest and agricultural fluxes (Fig. 2a) are shown in Fig. 6b. The prediction, based on Eq. 2, is dimensionless and the focus is on relative trends of the differences (i.e., the shape of the curve). As in the composited observations for 2013–2018 (Fig. 6a), the predicted pattern was low from January through April. In May, Tower 01 CO_2_ was predicted to be its greatest magnitude lower than Tower 09 CO_2_. This is attributable to forest drawdown in the area surrounding Tower 01. Leaf-out usually occurs in the Morgan-Monroe Forest south of Tower 01 beginning at the end of April and is 80% complete by the month of May [35], compared to agricultural drawdown which begins later in the year, June for corn and July for soybean (Fig. 2a). This conclusion is consistent with large enhancements noted when the wind was from the south in Additional file 1: Fig. S3c. The predicted pattern in background tower differences (Fig. 6b) was similar to the observed minimum (Fig. 6a) in June, but slightly ahead in time. The Tower 09 CO_2_ was predicted to be its greatest magnitude lower than Tower 01 in mid-July, only a 2-week shift from the observed pattern. That is, by mid-July, the agricultural fluxes in the footprint of Tower 09 were maximally larger than the forest fluxes in the footprint of Tower 01. While the predicted CO_2_ difference was small in November and December, as observed, the prediction indicated an additional minimum in September (about one half the magnitude of the minimum in May) that was not observed in the tower data. According to the fluxes in Fig. 2a, the forest was expected to continue to draw down CO_2_ in September, whereas the agricultural fluxes indicated small magnitude respiration. The tower results, however, indicated the both forest and agricultural fluxes were roughly balanced by mid-September, averaged over the time period of the dataset.

The forward model using VPRM and Hestia fluxes predicts an observed negative difference between Towers 09 and 01 in May and June and was largely consistent with observed differences throughout the rest of the year, but missed the positive difference in August and September (Fig. 6c). For May–mid June, the differences were − 1.0 ppm, Then, as for the prediction based on simple fluxes and landcover types, the mismatch switches sign and averages + 0.9 ppm for mid June–mid July. In the later part of the summer, August through mid-September, the model predicts the towers to have nearly the same CO_2_, missing the + 3.0 ppm observed difference. Improvements in the biogenic model can minimize these differences. Additionally an inversion using CO_2_ and CO mole fractions can optimize both the fossil and the biogenic CO_2_ separately [52].

#### Differential footprints

**Fig. 7:**
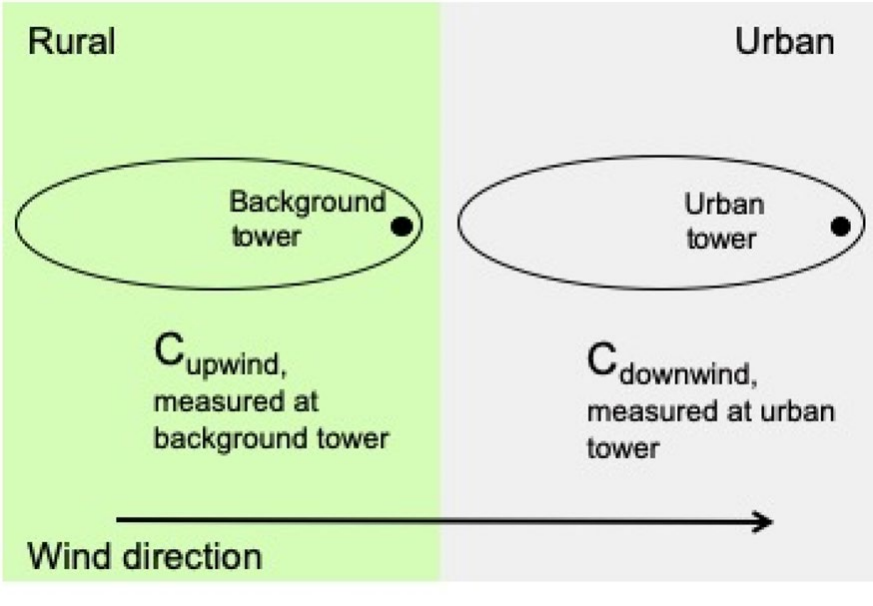
Schematic illustrating the “differential footprint” concept, as opposed to a simple box model. The green area indicates rural landcover surrounding the background tower and the gray area indicates urban landcover. The ellipses indicate the areas contributing the majority of the signal for each tower, since the influence decreases exponentially with distance from the tower. 80% of the influence for the INFLUX towers is within 10 km, on average, and for example, Towers 01 and 02 are separated by 43 km

A box model assumes that the species of interest measured downwind is that measured at an upwind location, changed only by the fluxes from the surface below the box. This approach is not appropriate for interpretation of the application described here because the influence function for each tower decreases exponentially with distance from the towers [43] and thus the tower footprints do not overlap to a large degree. Here we have considered one background regardless of wind direction in an Eulerian framework (ignoring wind direction for which either background tower was within the urban plume), but biological fluxes must be considered for more Lagrangian approaches (following an air parcel from a background tower to an urban tower) as well. Using a wind-direction-dependent background while still excluding urban plumes for the background towers yielded similar results in terms of predominant growing season enhancement not likely to be attributable to changing anthropogenic emissions (Additional file 1: Fig. S4c). Contributions to the CO_2_ measured at a tower decrease exponentially with distance (e.g., [43]. The footprints for a background and an urban tower overlap, but each tower is influenced preferentially by nearby sources, and we call this the differential footprint. If we consider a contour containing a given percentage of the influence, we can visualize the differing effect of the rural biological flux as in Fig. 7.

#### Modelled urban-tower averaged CO_2_ enhancements

**Fig. 8:**
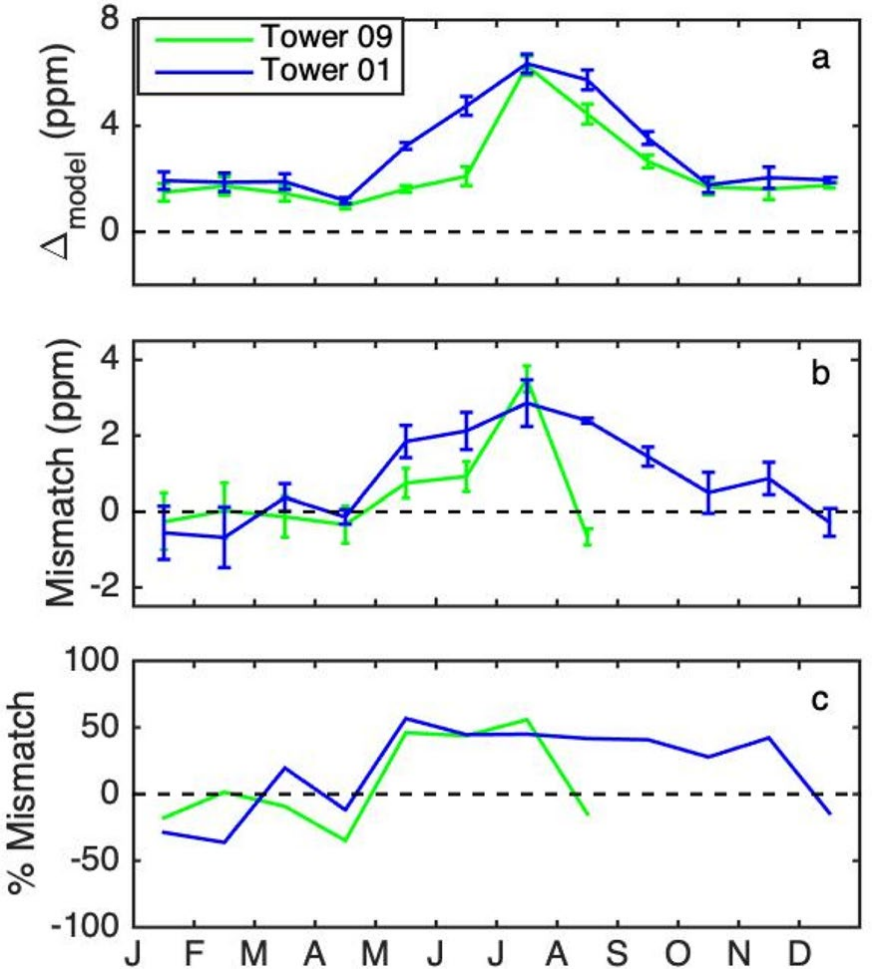
**a** Modelled CO_2_ enhancement using Tower 09 (agricultural) as a background (green) and Tower 01 (forested) as a background (blue), averaged over INFLUX urban towers for 2014. Wind directions for which either Tower 01 or Tower 09 are in the urban plume have been excluded. Only afternoon hours (1200–1700 LST) are included. Error bars indicate the standard error amongst the urban towers. **b** Model-data mismatch. Note that there was an instrument failure at Tower 09 for September–December 2014. **c** Percent mismatch, i.e., model-data mismatch divided by the modelled CO_2_ enhancement

The forward model predicted a growing season increase in enhancement in CO_2_ at the tower sites (Fig. 8a). Furthermore, the model was able to capture the observed timing difference in green-up between using forested Tower 01 and agricultural Tower 09 as backgrounds, in general. However, modelled enhancements were larger than observed (Fig. 5) during the growing season. The modelled difference between the CO_2_ of the background towers was similar to that observed in June (2.9 ppm modelled, compared to the observed 2.5 ppm), but in August, the model predicted the forested background tower CO_2_ to be lower than the agricultural tower by 1.7 ppm, whereas the observations showed the agricultural tower CO_2_ to be lower by 2.0 ppm.)

The INFLUX urban tower-averaged (Towers 02, 03, 06, 07, and 10) model-data mismatch, resulting from a combination of differences attributable to fossil fuel fluxes, biogenic fluxes and transport, was larger during the growing season (May–September) compared to the dormant season (Fig. 8b). For the dormant season, the mean model-data mismatch was − 0.1 ± 0.5 ppm. The mean growing season model-data mismatch was 1.1 ± 1.7 ppm for enhancements compared to Tower 09 (agricultural) and 2.1 ± 0.5 ppm for those compared to Tower 01 (forested), with the standard deviations calculated over the months. The modelled enhancements were positive in the growing season, likely indicating stronger modeled drawdown at the background sites than was observed, with the mismatch being higher for the forested background site. The INFLUX tower-averaged model-data mismatch averaged 25% of the modelled CO_2_ enhancement for Tower 09 and 29% for Tower 01 during the growing season.

#### Agricultural tower CO_2_ background differences

**Fig. 9:**
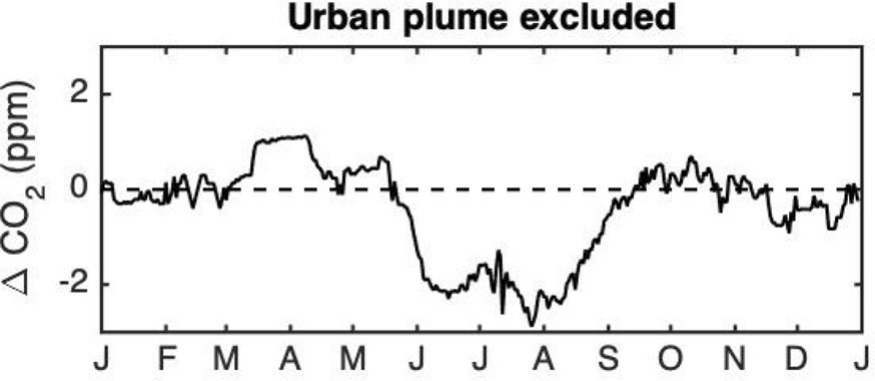
Composited 31-day running median CO_2_ differences between Towers 09 and 14, both agricultural background towers. As for the previous results, data for which Tower 01 or Tower 09 was influenced by the urban plume were excluded from the analysis (WSW and NE). Tower 14 was not significantly affected by the urban plume

The median daytime CO_2_ at Towers 14 and 09 differed by up to 2.5 ppm in the peak growing season, despite both being agricultural sites (Fig. 9). Tower 14, in west-central Indiana is on the eastern edge of the highly productive U.S. corn belt (Additional file 1: Fig. S5). Tower 14 is located in Montgomery County, which produced 1.35 × 10^6^ kg corn/harvested km^2^, whereas Hancock County (Tower 09) produced 1.06 × 10^6^ kg corn/harvested km^2^ [48]. In addition, the area surrounding Tower 14 contains 37.1% corn landcover (“CO_2_ enhancements above background” section), whereas the area surrounding Tower 09 is 33.8% corn. Thus the combination of a higher percentage of corn coverage and more productive harvests is likely to have contributed to the additional CO_2_ drawdown observed at Tower 14 compared to Tower 09.

We explored other potential causes, but none seem likely to explain the large observed differences between these two towers. Measurement height does likely contribute, but not enough to explain the difference (see also Section S4). The measurement heights of the three potential background towers (Towers 01, 09, and 14) are 121, 130, and 76 m AGL, respectively. Linearly interpolating the mean CO_2_ difference found between tower heights in the growing season at Tower 01 (Fig. S6), the magnitude of the difference between 76 m AGL and 121 m AGL was 0.3 ppm. This analysis is an overly conservative estimate since vertical CO_2_ gradients are non-linear [58]. Typical corn fluxes in July and August are about double those of forest (Fig. 2a), so the height effect difference between Tower 14 and Tower 09 is estimated to be no more than 0.6 ppm.

Although we ignored wind directions for which the primary plume from Indianapolis affected the background towers, there are still small urban areas within the footprints of the towers. The urban fraction within the 10-km^2^ area contributing about 80% of the influence on the CO_2_ measurements at Tower 09 was 12.3%, compared to 6.2% surrounding Tower 14. Thus increased anthropogenic signal at Tower 09 may have somewhat reduced the apparent biological signal, increasing the difference between Tower 14 and Tower 09. Tower 14 is outside the modeling domain, and thus has not been considered extensively in this work.

## Discussion/conclusions

We examined CO_2_ enhancements as a function of time throughout the year using composites of over six years of data from towers in and around the city of Indianapolis, IN. Three possible background towers were considered, Tower 01 in a forested area southwest of the city, Tower 09 in an agricultural region east of the city, and Tower 14 in an agricultural region northwest of the city. The enhancement differed significantly depending on choice of background and time of year, being 2.8 ppm higher in June and 1.8 ppm lower in August using Tower 01 as a background compared to Tower 09.

The most striking feature in the CO_2_ enhancements compared to agricultural Tower 09 as a background was an apparent maximum in August, with 31-day median enhancement at the urban towers up to 4.3–6.5 ppm, 2.6 times as large as those in the dormant season. This feature could be misinterpreted solely as an anthropogenic signal, but the cause was a combination of the effect of the biological signal upwind of the background tower, and a secondary maximum in the fossil fuel flux (Fig. 2b). Clearly the biological fluxes (and land cover types) upwind of the background measurement sites must be known in order to interpret CO_2_ enhancements throughout the year. Anthropogenic fluxes resulting from an inverse estimation which did not consider biological fluxes in the rural areas around the city would overestimate the summertime anthropogenic fluxes. We note that using a model-data hybrid approach for determination of background [16] minimizes the effect of the biogenic signal in the calculated enhancements. Our approach, using a measured background, means that the enhancements are quite dependent on the biogenic signal of the background towers, and the enhancements are not “anthropogenic” enhancements. In the next step, optimization of fluxes using an inversion [52], the biogenic portion is determined via either method of background determination. While we have focused on the biogenic signal at the background towers, urban biogenic CO_2_ fluxes also change with season [59]. CO_2_ drawdown by the urban biosphere affects the CO_2_ enhancements at urban towers compared to background towers, and is important to understand.

The difference in timing of fluxes from the different land cover types of the background towers appears to explain the shift in summertime peak for the urban towers from August when using agricultural Tower 09 as a background to July when using forested Tower 01 as a background.

While the differences in the growing season enhancements using different backgrounds were sizeable, a prediction of the difference between Tower 09 and Tower 01 CO_2_ based on differences in land cover type in the surrounding areas and the typical fluxes of these land cover types yielded a plausible explanation, with the difference attributable to the forest green-up preceding that of agriculture, but the agricultural peak drawdown being more intense. Forward modelled total CO_2_ using Hestia fossil fuel emissions and VPRM biogenic fluxes show that the biogenic model was able to represent the enhancements fairly well, with model-data mismatch of 1.1 ± 1.7 ppm for the agricultural background and 2.1 ± 0.5 ppm for the forested background during the growing season (25–29% of the modelled CO_2_ enhancement) and − 0.1 ± 0.5 ppm during the dormant season. Developing and testing robust CO_2_ flux estimates for the rural ecosystems upwind of cities is therefore critical to year-round urban anthropogenic CO_2_ flux estimates. Further tuning of the biogenic model response, or a more advanced vegetation model in order to more fully capture the timing and productivity differences between the forested and agricultural sites considered here would likely improve the inversion results. The sensitivity of the inverse fluxes to the biogenic fluxes is of course dependent upon the fossil fuel emissions for the study area.

The growing season forward model to data mismatch was larger than the dormant season mismatch, suggesting that biogenic fluxes were a larger source of mismatch than the fossil fuel fluxes. The inversion of these data, separately optimizing fossil fuel and biogenic CO_2_ emissions, [52] indicated very little adjustment to the fossil fuel CO_2_ emissions from Hestia. While the larger uncertainty assigned to the prior biogenic flux may have played a role, this result further indicates that the biogenic model is a larger contributor to the model-data mismatch. Unfortunately, we were not able to use the flask measurements to decompose into biogenic and fossil fuel components of the mismatch because (1) the flasks were sampled when the winds were from the west/southwest, i.e., to a large extent, the wind directions ignored for this analysis, and (2) differing subset of towers for this analysis vs the flask availability. In the future, a flask sampling strategy including wind directions for which neither background tower is in the urban plume would provide further evidence.

The summertime increase in enhancement was larger in forward model results than is observed, indicating that the VPRM fluxes were in general too strong, or that the modelled biosphere was too weak within the urban domain. Further analysis will assess the performance of VPRM via flux towers and tune the biogenic model to improve accuracy. Accurate modelling of ecosystems will be crucial for accurate fluxes during the growing season, for both the approach presented here, based on a simple background tower or wind-direction dependent tower, and the model-data hybrid approach for background determination [16]. Another approach is to optimize the biogenic fluxes separately in the inversion [52].

Enhanced intensity of drawdown due to corn during the peak growing season months of July and August northwest of Indianapolis was the likely cause of the large difference in CO_2_ measured at Towers 09 and 14, both in agricultural areas. The discrepancy between background agricultural sites during the peak growing season months was similar in magnitude to the differences between urban towers and Tower 01. Tower 14 is on the predominately downwind edge of the U.S. corn belt, and while there is corn grown in the area surrounding Tower 09 it is a less productive area overall. Persistent differences in CO_2_ between two background sites with similar land cover presents an additional challenge for vegetation models. The biogenic model may need to be further tuned to capture the differences between these agricultural sites with differing productivity. INFLUX is unique being on the edge of the U.S. corn belt, but in general, potential gradients in production and differing landcover type within domains for each study should be considered. Additional measurements, including flux tower eddy covariances are planned to learn more about the differences in CO_2_ drawdown between these locations.

Although the number of towers and timespan of the dataset is unprecedented for a study of CO_2_ mole fractions in and around a city, there were some limitations of this study. We used afternoon-averaged wind direction at the airport to exclude the afternoons for which the background towers were affected by the urban plume. Given the likelihood of wind direction changes throughout the day, back trajectory analysis would have been a more accurate way to exclude the urban plume, but is beyond the scope of this study. Additionally, although we estimated the effects of variable tower heights on our results and found them to be negligible (Additional file 1: Fig. S7), ideally all measurements would be made at the same height. In practice, this was not possible. Furthermore, we used a 10-km radius to categorize land cover types for each tower for the simple calculation shown in Fig. 5c. In reality, the area affecting the CO_2_ measured at each tower is much more complex and we did not address the seasonal cycle of the urban biosphere for the simple calculation. We have addressed these issues to a degree by comparing the observed enhancements to the forward modelled CO_2_. The modelled footprints were calculated on the inner, 1-km resolution, 87 km × 87 km domain, and likely extended beyond this domain. Future analysis will include footprints calculated on a larger domain.

Here we focus on the afternoon hours as these data are typically used in inversions. Afternoon is typically more well-mixed, allowing for less complicated interpretation and high fidelity modeling results. There is, however, critical information available during other times of day. Future analyses of nighttime respiration and the use of non-afternoon greenhouse gas data in inversions are likely to prove beneficial to understanding of the carbon cycle and reducing uncertainties.

This study in general highlights the importance of background choice in urban greenhouse gas studies. The magnitude of potential background differences depends on time of year and land cover types in the region. Indianapolis is a large city, but not a mega city, and determination of fluxes for larger cities with larger CO_2_ fluxes may be less affected by land cover-based differences in background towers. Still, careful consideration of land cover types is necessary in order to interpret CO_2_ tower network data throughout the growing season. Each city, depending on topography, climate, population, surrounding land cover and other factors, has unique challenges for the estimation of greenhouse gas emissions.

## Supplementary information

**Additional file 1: Figure S1.** Afternoon average CO_2_ time series for the background towers. **Figure S2.** Hourly-averaged CO_2_ for the six specific days with the largest deviations amongst the potential background towers. **Figure S3.** Afternoon-average CO2 differences of background towers as a function of wind direction and season. **Figure S4.** Composited 31-day running median afternoon-average CO_2_ enhancements using alternate backgrounds. **Figure S5.** Corn production by county for 2017. **Figure S6.** Composited afternoon CO_2_ vertical profiles for growing and dormant seasons. **Figure S7.** Composited median afternoon-average CO_2_ enhancement from Tower 01, as a function of urban land cover fraction and tower height.

## Data Availability

Miles NL, Richardson SJ, Davis KJ, Haupt BJ. In-situ tower atmospheric measurements of carbon dioxide, methane and carbon monoxide mole fraction for the Indianapolis Flux (INFLUX) project, Indianapolis, IN, USA. Data set available on-line from The Pennsylvania State University Data Commons, 2017b; http://dx.doi.org/10.18113/D37G6P. For further information, see http://sites.psu.edu/INFLUX.
