# Supplementary material for: The influence of near-field fluxes on seasonal carbon dioxide enhancements: results from the Indianapolis Flux Experiment (INFLUX)
Source: Carbon Balance Manag. 2021 Jan 30;16:4. doi: 10.1186/s13021-020-00166-z (PMC7847578; doi:10.1186/s13021-020-00166-z)
Supplement: Supplementary file 1 — Additional file 1: Figure S1. Afternoon average CO2 time series for the background towers. Figure S2. Hourly-averaged CO2 for the six specific days with the largest deviations amongst the potential background towers. Figure S3. Afternoon-average CO2 differences of background towers as a function of wind direction and season. Figure S4. Composited 31-day running median afternoon-average CO2 enhancements using alternate backgrounds. Figure S5. Corn production by county for 2017. Figure S6. Composited afternoon CO2 vertical profiles for growing and dormant seasons. Figure S7. Composited median afternoon-average CO2 enhancement from Tower 01, as a function of urban land cover fraction and tower height. [file 13021_2020_166_MOESM1_ESM.docx]

**Additional file**

In this document, we discuss 1) differences in background site CO_2_ as a function of time and wind direction, 2) enhancements compared to alternate backgrounds, 3) observed and modelled CO_2_ enhancements, 4) corn production, and 5) tower height effects.

**Differences in background site CO_2_
Background CO2 differences as a function of time**

In general, the CO_2_ time series for the three potential background towers (including all wind directions) was more variable in the summer months of July and August (Fig. S1a), due to the drawdown and respiration of the biosphere. Differences between the background sites were as large as 18 ppm (Fig. S1b). Examination of the diurnal cycles of the nine days in January – August 2018 with largest differences (Fig. S2) showed that the differences were attributable to the urban plume in most cases.


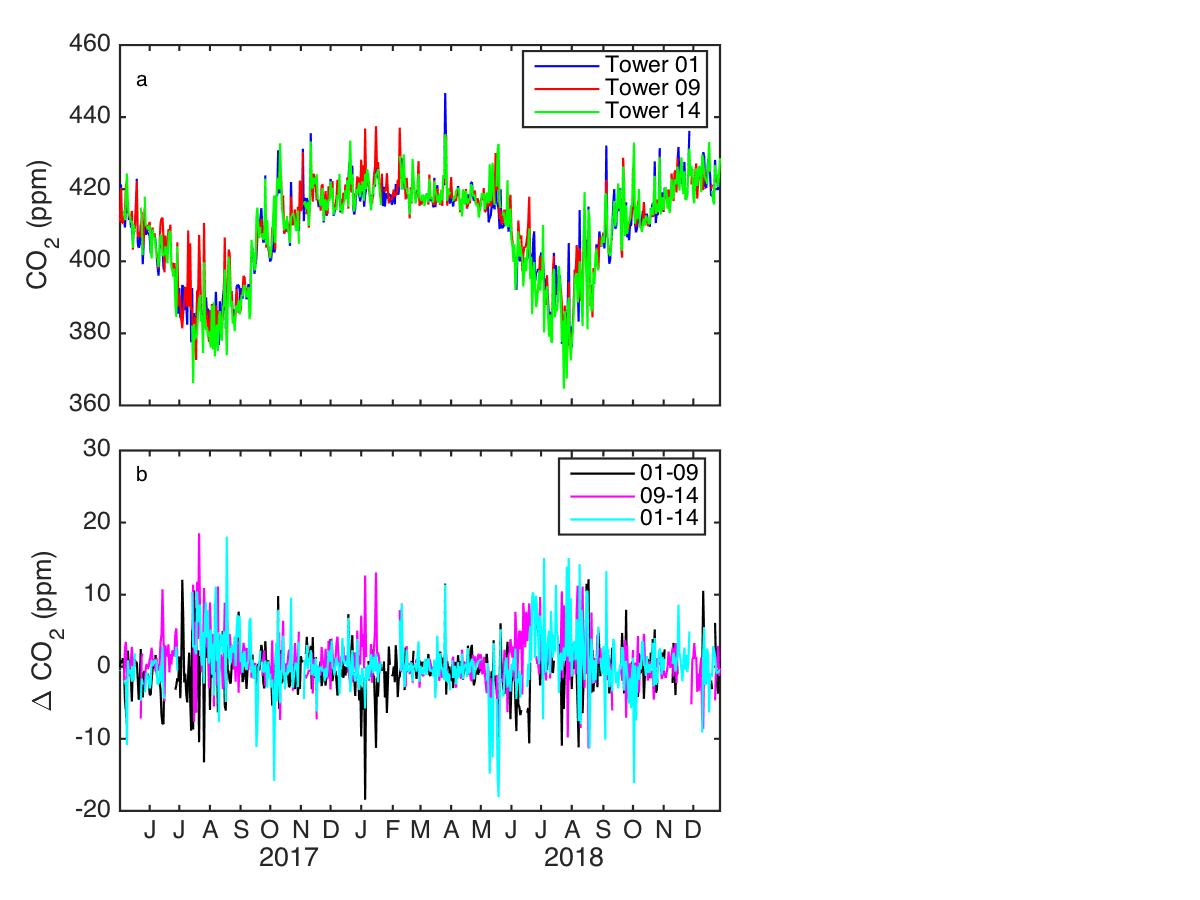


Figure S1. (a) Afternoon average CO_2_ as a function of time for May 2017 – December 2018, for background towers (Towers 01, 09, and 14). (b) Afternoon average CO_2_ difference between pairs of potential background towers, with all wind directions included. Tickmarks indicate the beginning of each month. Note that these plots include Tower 14 data for 26 April – 5 November 2017, a period with increased measurement uncertainty which are excluded from further analysis.

No evidence of differences in the timing of ABL growth between the towers was noted through examination of the hourly average values as a function of time of day for several examples of days with large inter-tower CO_2_ differences (Fig. S2).


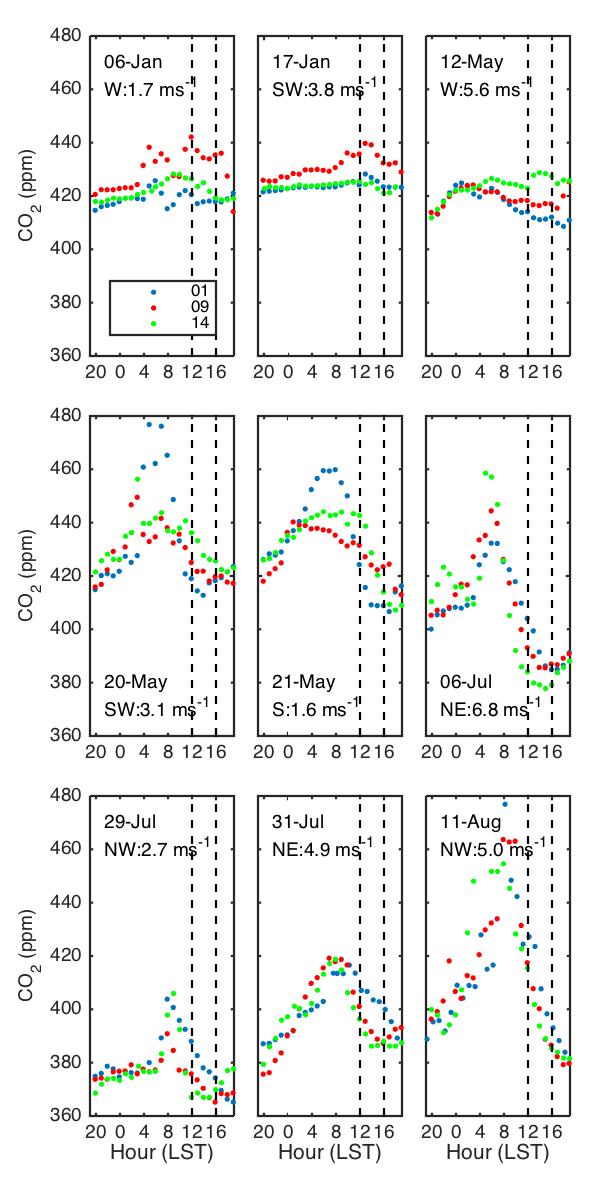


Figure S2. Hourly-averaged CO_2_ for the six specific days with the largest deviations amongst the potential background towers for Tower 01 (blue), Tower 09 (red) and Tower 14 (green). Vertical dashed lines indicate 1200 and 1600 LST, the period over which the afternoon averages were determined. The date (in 2018) and afternoon-averaged wind direction and wind speed are indicated in each plot.

**Background CO_2_ differences as a function of wind direction
Tower 09 compared to Tower 01**

During the dormant season (January/February), ABL CO_2_ mole fraction differences between Towers 01 and 09 are dominated by the urban plume. The differences between background Towers 01 and 09 as function of afternoon average wind direction and speed are shown in Fig. S3a,c,e. For January and February for the years 2013–2018, the CO_2_ measured at Tower 09 was 0.7 ppm higher than that at Tower 01_,_ averaged over all wind directions. Examination of the differences as a function of wind direction revealed the effects of the city. The highest positive differences for these dormant season months were for wind directions from the NE, when Tower 01 was in the urban plume and measured 2.1–2.8 ppm CO_2_ above that of Tower 09 (Fig. S3a). For the purposes of excluding the urban plume for subsequent analyses, we excluded wind directions between 20 – 65° for Tower 01, encompassing the large positive signals. When the wind direction was from the WSW, Tower 09 was in the urban plume, and the median Tower 09 CO_2_ mole fraction was higher than that at Tower 01 by 1.7 ppm. We considered wind directions between 235 – 280° as those for which Tower 09 was affected by the urban plume. Although the differences for wind directions from the ESE and SSW were 1.6 and – 1.1 ppm, respectively, the wind directions were not such that either tower was downwind of the urban area. For the remaining wind directions, from the northwest and the southeast, the median differences were less than or equal to 0.3 ppm in magnitude or not significant compared to the standard error. We note that while Miles et al. (2017a) reported evidence of a CO_2_ source to the SSE of Tower 01 during the period 1 January – 20 April 2013, we did not see such evidence when considering the increased statistics from multiple years of data.

During late spring (May/June), forest phenology causes the daytime ABL CO_2_ mole fraction at Tower 01 to be persistently lower than the mole fractions observed at Tower 09 (Fig. S3c). Tower 01 CO_2_ mole fractions were lower than Tower 09 by 1.7 ppm averaged over all wind directions. The largest median differences between Towers 09 and 01 occurred when the wind was broadly from the south, with median differences between 1.6 and 4.2 ppm. This pattern is consistent with seasonal timing differences in drawdown due to different land cover types at Tower 01 (largely forested) compared to Tower 09 (largely agricultural) and typical seasonal patterns in fluxes of those land cover types (Fig. 3). By May, leaf-out has typically occurred in the forested region south of Tower 01 (Kim et al. 2015), causing CO_2_ drawdown at Tower 01 and larger magnitude negative differences compared to Tower 09. Agricultural drawdown is not maximized until July (Hollinger et al. 2005). When Tower 09 was in the urban plume, the differences were also large, up to 2.4 ppm. From other directions NNW through WSW, the magnitude of the median differences was small compared to the standard errors. The effect of the urban plume at Tower 01 (when wind directions were from the NE) were not apparent, unlike during the dormant season months.

During the peak growing season (July/August), the drawdown from the forest to the south of Tower 01 is not as apparent as the agricultural signal from the corn and soy surrounding Tower 09. The average difference over all wind directions switched in sign compared to May/June, and Tower 09 measured 1.4 ppm lower CO_2_ than at Tower 01. For wind directions from the WSW when the urban plume affected Tower 09, the median Tower 09 CO_2_ was higher than that at Tower 01 by up to 2.9 ppm for July/August (Fig. S3e), compared to 1.7 ppm higher from that direction in the dormant season. From wind directions broadly easterly, the median Tower 09 CO_2_ was lower than Tower 01 by up to 4.5 ppm.


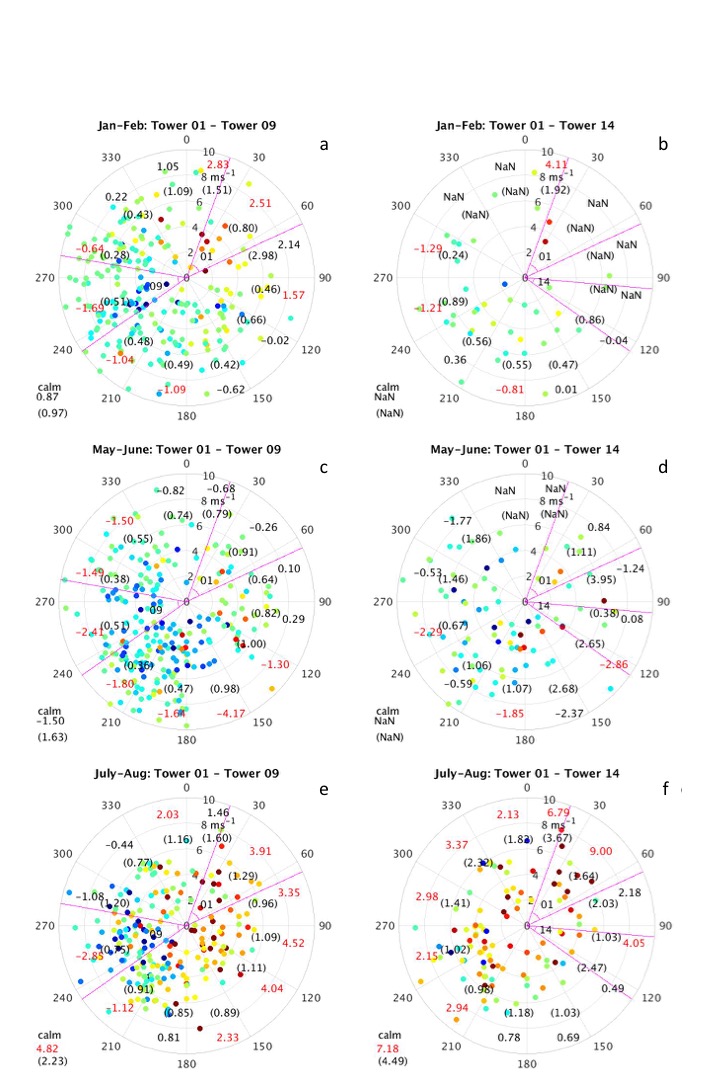


Figure S3. Afternoon-average CO_2_ differences of Tower 09 from Tower 01 (a,c,e) and of Tower 14 from Tower 01 (b,d,f) as a function of wind direction (angle) and wind speed (radial distance) for various time periods. (a, b) For dormant season, January – February for the available years 2013 – 2018. The median CO_2_ mole fraction within each wind direction range is indicated on the plots. The standard error for each wind direction range is indicated in parentheses. (c, d) For early growing season, May – June. (e, f) For late growing season, July – August. Magenta lines indicate the wind directions for which Towers 09 and 01, and Towers 14 and 01, respectively, were directly downwind of Indianapolis. Medians larger than the magnitude of the standard error by the measurement uncertainty (0.2 ppm) or larger are indicated by red numbers. Wind directions for which there are fewer than three days in the appropriate period are indicated by ‘NaN’. For time periods with three or more afternoons with calm winds (<1.6 ms^-1^), medians and standard errors are indicated in the lower left corner of each plot. Note that these plots include Tower 14 data for 26 April – 5 November 2017, a period with increased measurement uncertainty which are excluded from further analysis.

Tower 14 compared to Tower 01

While both Towers 01 and 09 were operational for the years 2013 – 2018, Tower 14 was deployed in 2017 as an additional background site. Thus there were fewer overall data points, as is particularly apparent when separated by wind direction (Fig. S3b,d,f).

On average over all wind directions, the median difference between Tower 14 and Tower 01 afternoon-average ABL CO_2_ mole fraction was 0.0 ppm for the months of January and February, but the differences varied with wind direction according to the location of urban emissions. The CO_2_ mole fractions observed at the two towers were not significantly different for most wind directions (Fig. S3b) during this period. The urban plume was not observed in the CO_2_ measured at Tower 14 (wind directions from the southeast). Tower 14 is 44 km from the edge of Indianapolis. Tower 14 measured higher CO_2_ with winds from the WNW and WSW by 1.2 – 1.3 ppm. The town of Crawfordsville is 13 km WNW of Tower 14. For the limited number of days for which the winds were from the NNE, Tower 01 was downwind of the city and measured 4.1 ppm higher CO_2_ than Tower 14.

As was apparent in the comparison between Tower 01 and Tower 09, the afternoon CO_2_ mole fraction at Tower 01 was lower than that measured at Tower 14 during May and June, coincident with drawdown from the forests south of Tower 01. Averaging over all wind directions, Tower 01 measured 1.1 ppm lower CO_2_ during these months. As for Tower 09 during May/June, Tower 01 measured lower CO_2_ than Tower 14 when wind directions were broadly southerly, specifically from SE to WSW, with median magnitude up to 2.9 ppm (Fig. S3d). When the wind directions were from remaining wind directions, the differences between the two towers was not significant compared to the standard error.

During the peak growing season months of July and August, crops (primarily corn and soy) played an important role in the CO_2_ patterns observed at the background towers. Tower 14 measured 3.4 ppm lower CO_2_ than Tower 01, with the difference for most wind directions being significant compared to the standard error (Fig. S3f). Larger differences are observed when the winds were aligned such that Tower 01 was downwind of the city, NNE and NE (up to 9.0 ppm). When the afternoon winds were calm, the median CO_2_ at Tower 14 was 7.2 ppm lower than that of Tower 01.

**Enhancements compared to alternate backgrounds**

Large growing season enhancement is apparent for various alternate background considered, including the more Lagrangian approach of utilizing a wind-dependent background (Fig. S4). Some differences are, however, notable. When Tower 01 is used as the background, the growing season occurs earlier in the year (mid June rather than the beginning of August for Tower 09 as a background) (Fig. S4a). This shift is consistent with the timing of the typical fluxes of predominant land cover types surrounding these towers (Fig. 3). As has been described (Section 3.3.4), the growing season drawdown is stronger at Tower 14 compared to Tower 09 and the growing season enhancement at the urban sites is thus larger with this tower used as a background (Fig. S4b). Increased growing season enhancement is also apparent when using upwind towers as background (an Lagrangian approach, but not lagged in time) (Fig. S4d,e). We note that the enhancements shown in Fig. S4d,e cannot be directly compared to those in Fig. S4a-c because of differing wind directions.


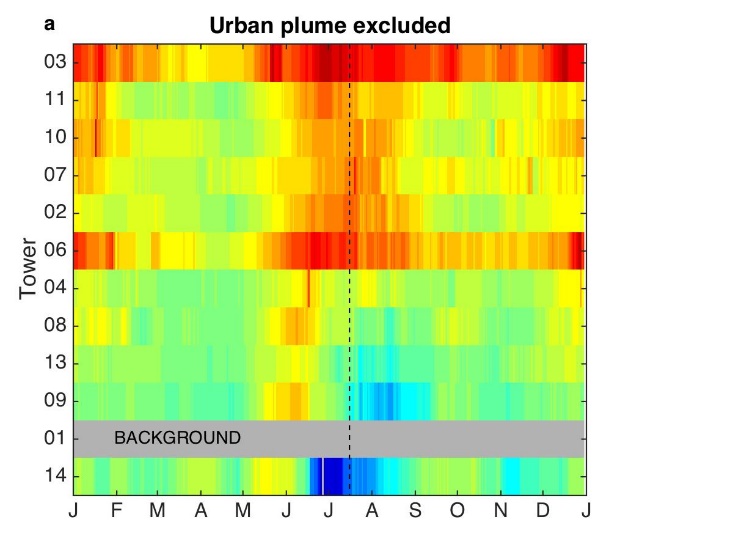

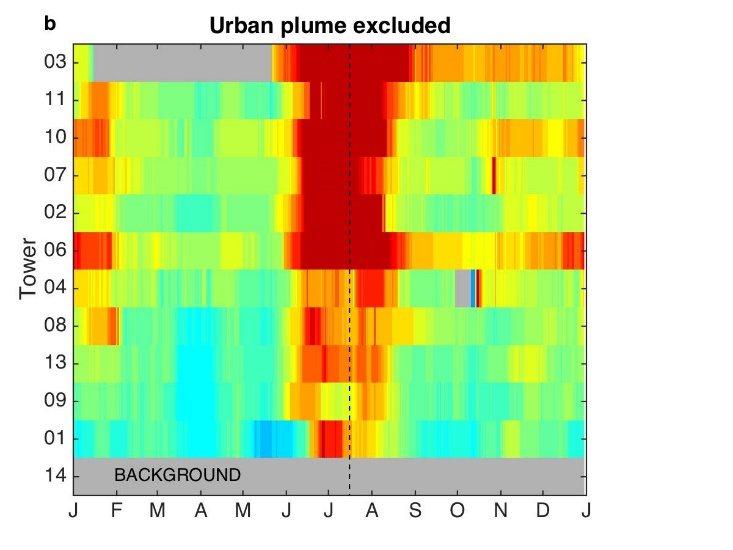


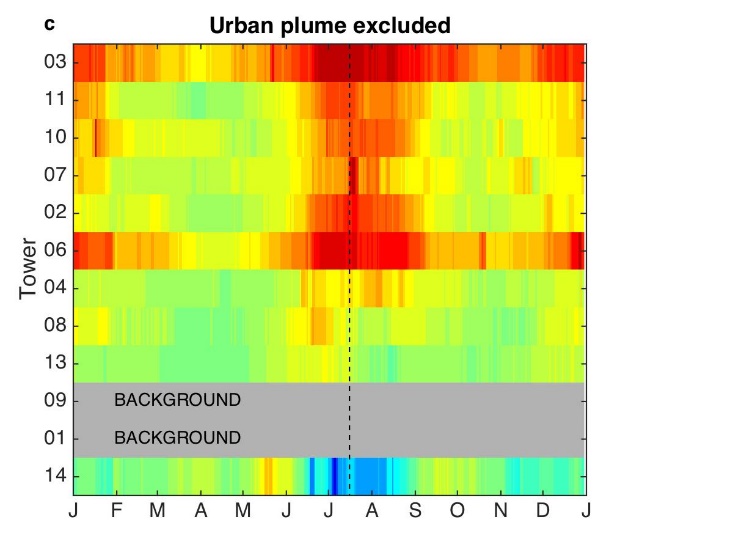

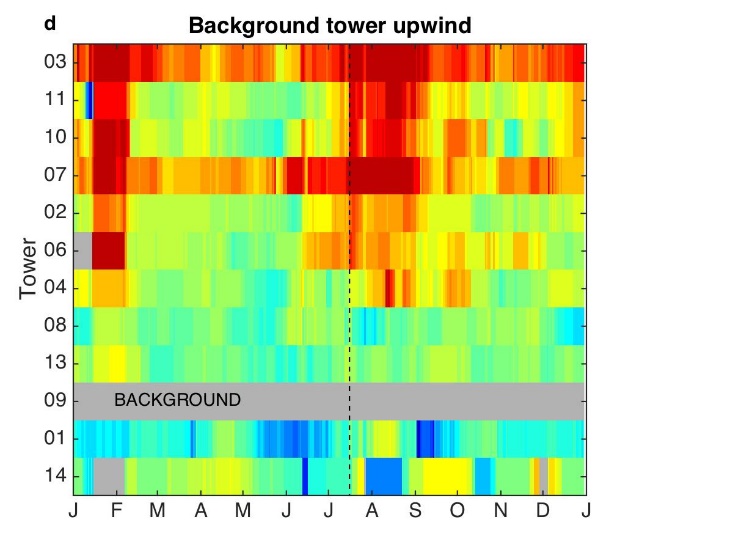

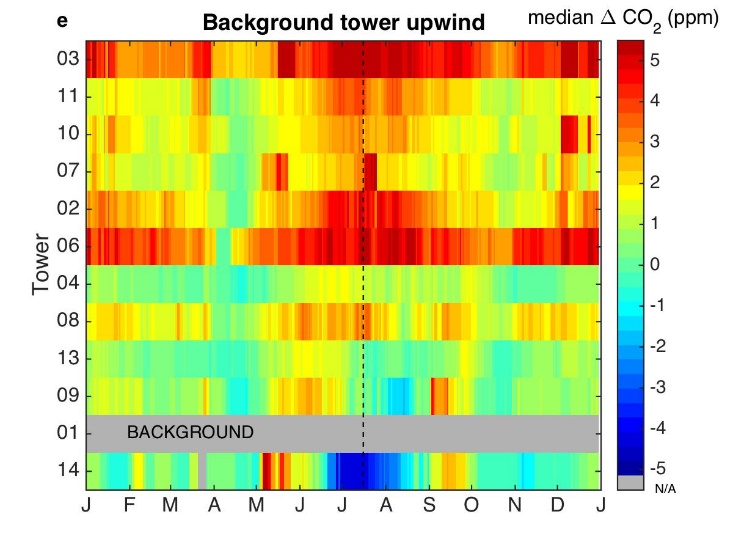


Figure S4. (a) Composited 31-day running median afternoon-average CO_2_ enhancements from Tower 01 for each of the towers using data from January 2013 through December 2018. The towers are ordered by urban fraction (including high-, medium-, and low-density urban land cover, as discussed in Section 3.1. Tickmarks indicate the beginning of each time period. Data for which Tower 01 or Tower 09 was influenced by the urban plume were excluded from the analysis (i.e., days with SW or NE winds are excluded). Dashed lines indicate July 15. Non-background towers deployed for less than 3 years are not shown (Towers 05 and 12). (b) As in (a), but enhancements from Tower 14. (c) Composited CO_2_ difference from a wind-direction dependent background (i.e., Tower 01 for wind directions between 180 – 360° and Tower 09 for 0 – 180°). To allow for comparison with a) and b), wind directions for which either background tower was affected by the urban plume are ignored. (d) Composited 31-day running median afternoon-average CO_2_ difference from Tower 09 calculated over moving 31-day windows for each of the towers, using data from January 2013 through December 2018, only when Tower 09 is upwind (55 – 100°). (e) As in (d), but composited CO_2_ difference from Tower 01, when Tower 01 is upwind of the city (210 – 240°). The enhancements for (a) – (c) cannot be directly be compared with those for (d) and (e) because of differing wind directions.

**U.S. corn production**

The INFLUX region is on the edge of the U.S. corn belt, as shown in the map of U.S. corn production by county (Fig. S5).


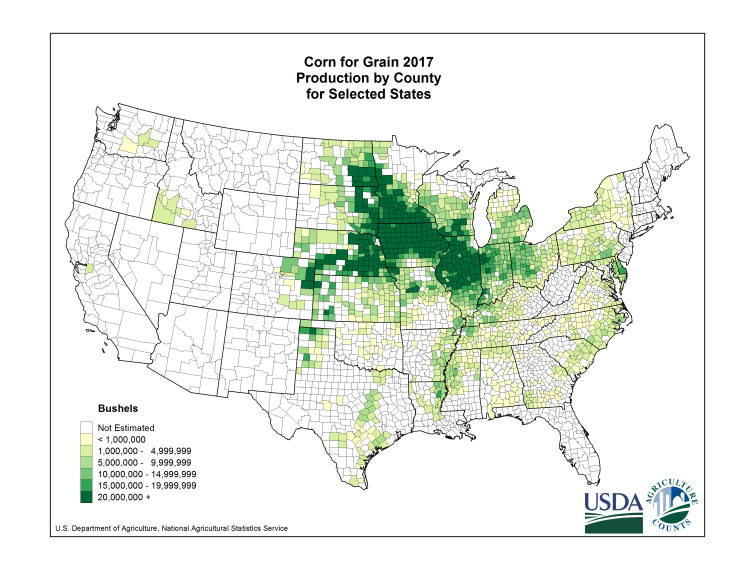


Figure S5. Corn production by county for 2017 (USDA NASS 2018).

**Tower height effect on urban and rural CO_2_ differences**

Here we consider the effect on tower height on the results for urban and rural CO_2_ differences. INFLUX towers 03, 10, 07, 06, 08, and 04 are 39 – 60 m AGL, whereas the remaining towers (including the background towers) are 87 m AGL or higher. The magnitude of the enhancement was certainly affected by the tower heights with the extent depending on the magnitude of the fluxes. For example, for a growing season period (1 June – 31 July 2013), the afternoon-only CO_2_ measured at the top level (121 m AGL) at Tower 01 was 0.5 ppm greater than that measured at 40 m AGL (Fig. S6). Conversely dormant season CO_2_ at Tower 01, with low fluxes at this background site, did not vary significantly with height. At Tower 03, the profiles were similar for growing and dormant seasons, and the CO_2_ at the top level (54 m AGL) averaged 0.8 ppm above the 40 m AGL measurement, a large gradient consistent with large anthropogenic fluxes near that site.


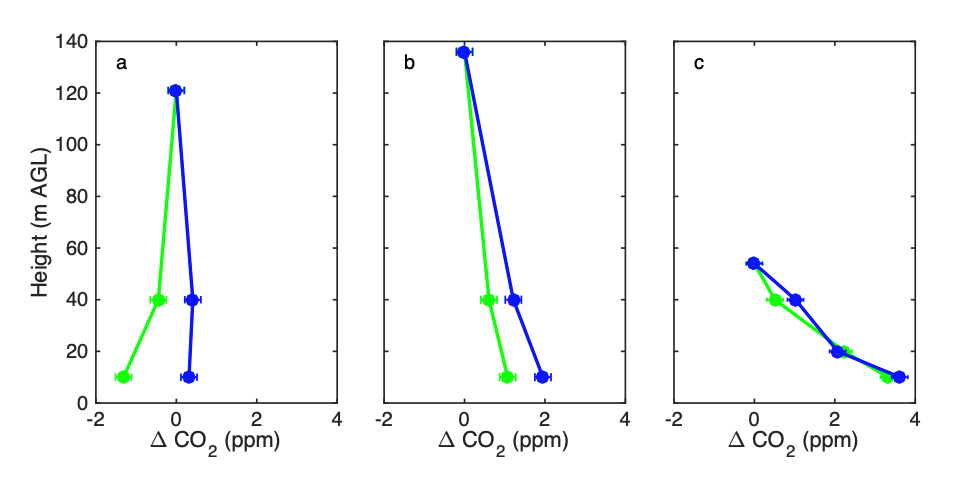


Figure S6. Composites of the difference between afternoon (1500–1600 LT) CO_2_ measured at each level and that of the top level for a) Tower 01, b) Tower 02, and c) Tower 03. Green dots indicate the average for two months during the growing season (1 June – 31 July 2013) and blue dots, for two months during the dormant season (1 November – 31 December 2013). Bars indicate the accuracy of the CO_2_ mole fraction measurements.

Despite the varying tower heights affected the enhancements, the predominant feature of large growing season enhancements is apparent regardless of tower height. The composited median CO_2_ enhancements compared to Tower 01 are shown as a function of urban fraction and tower height in Fig. S7. For dormant season months of January/February (Fig. S7a), the demarcation between urban and rural towers is clear, despite differences in tower heights. Amongst the rural sites, there was a correlation between increasing CO_2_ (primarily anthropogenic) and increasing urban fraction (r = 0.78, p = 0.12 so 12 % probability of this correlation occurred by random chance). There was not a significant correlation between CO_2_ and tower height (r = 0.40, p = 0.50). Tower heights and urban fraction were weakly correlated (r = 0.70, p = 0.19) for the rural sites, so completely deconvolving these effects was not possible.

The correlations between tower height, urban fraction, and median CO_2_ enhancements were all low (r = 0.41 – 0.50 and not significant) for the urban sites considered as a group. We also note that the median enhancements at Tower 03 (54 m AGL) during January/February were 4.3 ppm, whereas those at Tower 11 (130 m AGL) were 2.1 ppm, despite similar urban fractions. The urban land cover fraction does not include information on the spatial distribution of CO_2_ sources and sinks or the predominant wind direction, and is not meant to be an exact predictor of CO_2_ flux or CO_2_ enhancements. The enhancements at Tower 03 are influenced by traffic emissions in the immediate vicinity of the tower.

For the peak growing season months of July/August, the separation between rural and urban sites was again apparent (Fig. S7b). If tower height were the dominant effect, we would expect agricultural towers with lower heights to measure lower CO_2_ (more negative when compared to Tower 01), but this was not the case. Instead the CO_2_ difference was dominated by urban fraction (associated with less biogenic signal).


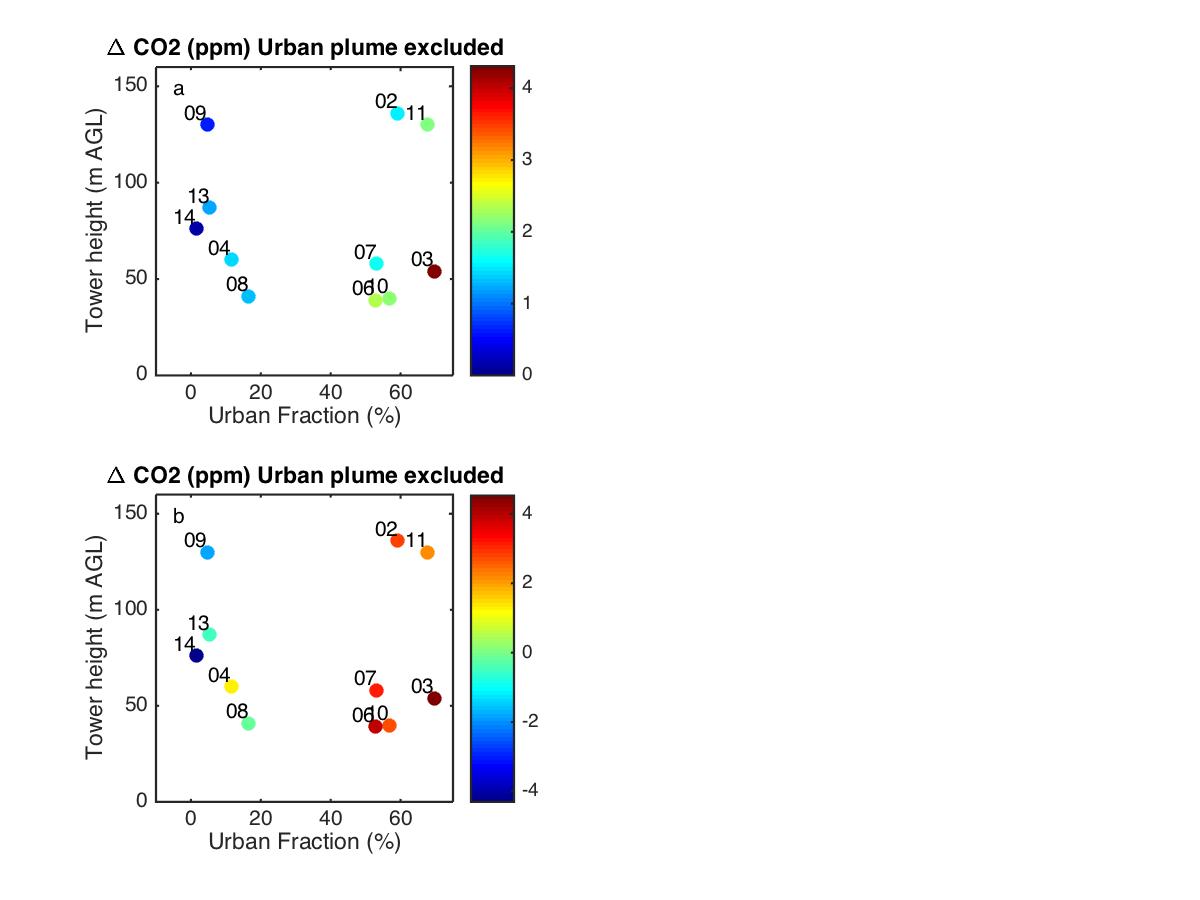


Figure S7. (a) Composited median (median for January/February) afternoon-average CO_2_ enhancement from Tower 01, as a function of urban land cover fraction and tower height. Data for which Tower 01 or Tower 09 was influenced by the urban plume were excluded from the analysis (i.e., only days with NW or SE winds were included). ‘Rural’ towers with less than 17 % urban fraction include Towers 08, 04, 13, and 14, whereas the ‘urban’ towers had greater than 50 % urban fraction. (b) Same as (a), but for July/August. Note the differing scales for (a) and (b).
